# Closed-Loop, Cervical, Epidural Stimulation Elicits Respiratory Neuroplasticity after Spinal Cord Injury in Freely Behaving Rats

**DOI:** 10.1523/ENEURO.0426-21.2021

**Published:** 2022-02-08

**Authors:** Ian G. Malone, Mia N. Kelly, Rachel L. Nosacka, Marissa A. Nash, Sijia Yue, Wei Xue, Kevin J. Otto, Erica A. Dale

**Affiliations:** 1 Department of Electrical and Computer Engineering, University of Florida, Gainesville, FL 32611; 2 Breathing Research and Therapeutics Center, University of Florida, Gainesville, FL 32611; 3 Department of Physical Therapy, University of Florida, Gainesville, FL 32611; 4 Department of Physiology and Functional Genomics, University of Florida, Gainesville, FL 32611; 5 Department of Biostatistics, University of Florida, Gainesville, FL 32611; 6 McKnight Brain Institute, University of Florida, Gainesville, FL 32611; 7 J. Crayton Pruitt Family Department of Biomedical Engineering, University of Florida, Gainesville, FL 32611; 8 Department of Materials Science and Engineering, University of Florida, Gainesville, FL 32611; 9 Department of Neurology, University of Florida, Gainesville, FL 32611; 10 Department of Neuroscience, University of Florida, Gainesville, FL 32611

**Keywords:** cervical spinal cord injury, diaphragm EMG, epidural stimulation, respiratory neuroplasticity, respiratory rehabilitation, spinal motor evoked potentials

## Abstract

Over half of all spinal cord injuries (SCIs) are cervical, which can lead to paralysis and respiratory compromise, causing significant morbidity and mortality. Effective treatments to restore breathing after severe upper cervical injury are lacking; thus, it is imperative to develop therapies to address this. Epidural stimulation has successfully restored motor function after SCI for stepping, standing, reaching, grasping, and postural control. We hypothesized that closed-loop stimulation triggered via healthy hemidiaphragm EMG activity has the potential to elicit functional neuroplasticity in spinal respiratory pathways after cervical SCI (cSCI). To test this, we delivered closed-loop, electrical, epidural stimulation (CLES) at the level of the phrenic motor nucleus (C4) for 3 d after C2 hemisection (C2HS) in freely behaving rats. A 2 × 2 Latin Square experimental design incorporated two treatments, C2HS injury and CLES therapy resulting in four groups of adult, female Sprague Dawley rats: C2HS + CLES (*n* = 8), C2HS (*n* = 6), intact + CLES (*n* = 6), intact (*n* = 6). In stimulated groups, CLES was delivered for 12–20 h/d for 3 d. After C2HS, 3 d of CLES robustly facilitated the slope of stimulus-response curves of ipsilesional spinal motor evoked potentials (sMEPs) versus nonstimulated controls. To our knowledge, this is the first demonstration of CLES eliciting respiratory neuroplasticity after C2HS in freely behaving animals. These findings suggest CLES as a promising future therapy to address respiratory deficiency associated with cSCI.

## Significance Statement

Every year, globally, 250,000–500,000 people sustain a spinal cord injury (SCI). Over half of these injuries occur in the upper cervical spinal cord, disrupting bulbospinal pathways to cervical respiratory neurons, which impairs breathing ability. This diminished respiratory function is the leading cause of death among spinal cord injured patients. We adapted a method, which partially restores walking in paralyzed humans, to electrically stimulate the cervical spinal cord below the level of injury concurrent with breathing rhythm. We hypothesize this cervical-spinal electrical stimulation induces spinal respiratory plasticity (i.e., promotes lasting beneficial changes to the spinal network responsible for respiration). Our findings support this hypothesis, establishing this method as a promising potential therapy to ameliorate respiratory complications after SCI.

## Introduction

Over half of the annual 17,000 new spinal cord injury (SCI) cases in the United States occur at the cervical level (McDonald and Sadowsky, 2002; Vinit et al., 2006; National Spinal Cord Injury Statistical Center, 2020), which can damage bulbospinal projections and disrupt breathing ability (Fuller et al., 2006; Vinit et al., 2006). Respiratory insufficiency remains the leading cause of morbidity and mortality following cervical SCI (cSCI), necessitating the development of therapies to restore respiratory function (Garshick et al., 2005; Waddimba et al., 2009; Krause et al., 2016; Hall et al., 2019). Diaphragm pacing and phrenic nerve stimulation are promising (Carter et al., 1987; DiMarco, 2005; Brown et al., 2006; Romero et al., 2012), and recent reports demonstrate that spinal motor circuits may be activated via intraspinal (Sunshine et al., 2018), epidural (DiMarco et al., 2006, 2020; Kowalski et al., 2013; Gonzalez-Rothi et al., 2017; Bezdudnaya et al., 2018), or temporal interference (Sunshine et al., 2021) stimulation, with open or closed-loop protocols (McPherson et al., 2015; Wagner et al., 2018). While these approaches show potential, many methods, unfortunately, require continuous stimulation and device dependence. Thus, it is imperative to develop rehabilitative strategies to reinstate sufficient and persistent breathing ability for meaningful improvements in the quality and duration of life after cSCI.

Epidural stimulation has remarkable potential to improve volitional sensorimotor and autonomic responses, even in cases of clinically complete SCI (DiMarco, 2001; DiMarco et al., 2006; De Vries et al., 2007; Ardell et al., 2009, 2014; Edgerton and Harkema, 2011; Edgerton and Roy, 2012; Tator et al., 2012; Angeli et al., 2014; Duru et al., 2015). Most (∼65%) SCIs have spared axonal projections traversing the injury site (Bunge et al., 1997). Much of the success of epidural stimulation in the lumbosacral spinal cord has been attributed, in part, to these inputs (Dimitrijevic et al., 1984; Sherwood et al., 1992, 1996; Edgerton et al., 2004; McKay et al., 2004; Rejc and Angeli, 2019). We predict that post-cSCI therapies targeting the cervical spinal cord can also benefit from spared fibers. However, rather than applying epidural stimulation techniques developed for the locomotor circuitry, factors specific to respiratory motor circuitry and the cervical spinal cord must be considered. For example, stimulation targeting locomotor networks likely relies on activation of an intraspinal circuit of pattern-generating neurons (Côté et al., 2018). The rhythm generators for breathing, however, lie within the ventral respiratory column of the brainstem (Smith et al., 1991), several segments rostral to the motor pools that they innervate and thereby are preserved after injury. Endogenous signals from intact rhythm generators provide the unique opportunity to harness activity-dependent mechanisms of neuroplasticity by stimulating respiratory motor pools in phase with a recorded rhythm (for review, see Malone et al., 2021). We predict using the breathing signal from intact components to time epidural stimulation in a closed-loop manner could be advantageous over open-loop paradigms (i.e., unpaired from respiratory rhythm), since (1) spinal respiratory circuits may be stimulated in a fatigue-resistant fashion; (2) stimulation will be endogenously adjusted to native respiratory load; and (3) the respiratory circuitry is highly plastic and could be “trained” via closed-loop reinforcement (e.g., Hebbian, activity-dependent plasticity).

**Figure 1.**
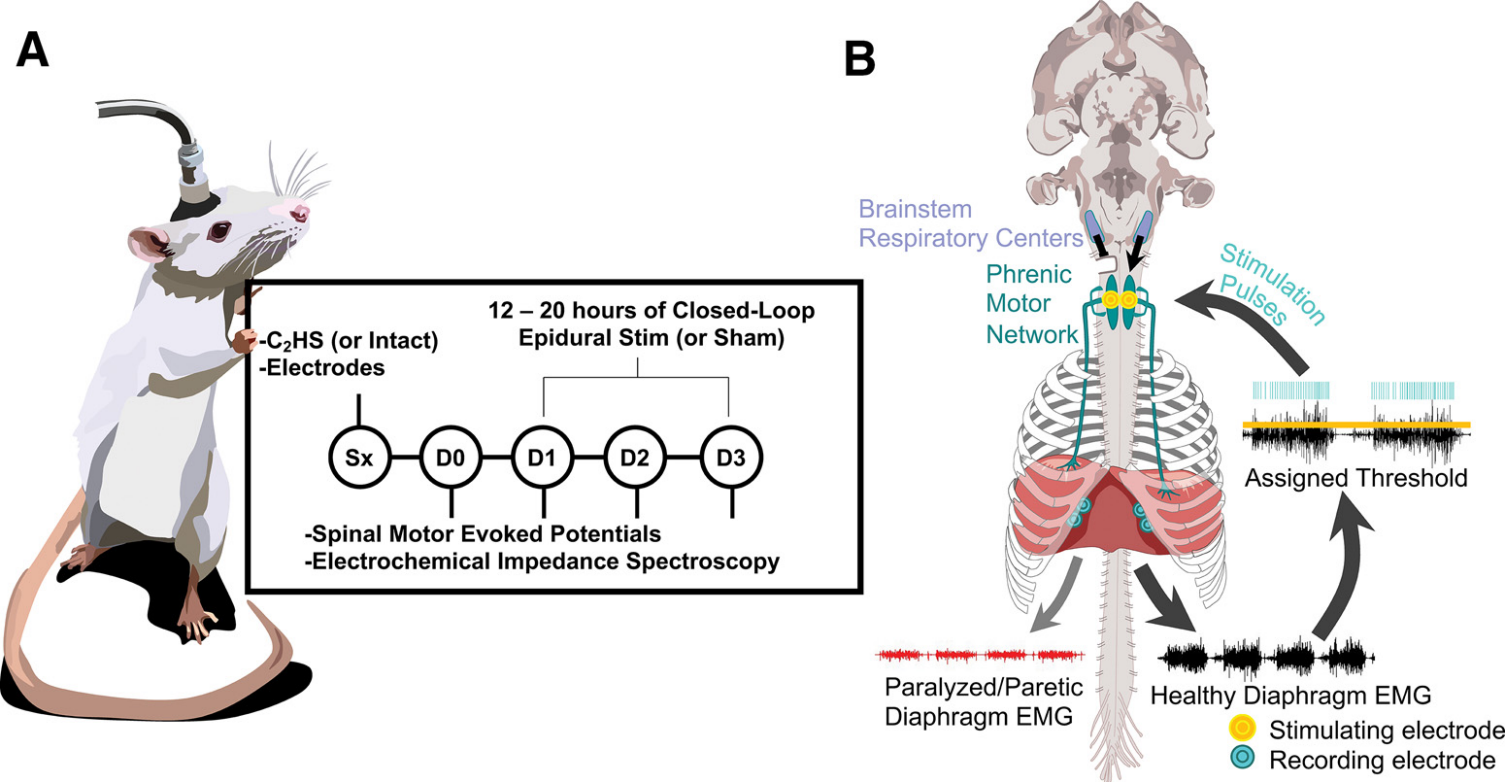
Experimental timeline and diagram of injury and implanted electrodes. ***A***, Rat shown with headcap and attached cable, which connects to recording and stimulation equipment. The headcap holds leads that are routed to implanted diaphragm recording and spinal epidural stimulating electrodes. On the surgery day, all rats are implanted with electrodes, and rats in appropriate groups receive a C2HS. On days 0, 1, 2, and 3, sMEP and EIS are measured. Between each day’s measurement, stimulated rats receive 12–20 h of CLES. ***B***, A C2HS disrupts ipsilesional descending bulbospinal inputs from brainstem respiratory centers (purple) to the phrenic motor pool (green) resulting in paralysis/paresis of the ipsilesional hemidiaphragm while the contralesional diaphragm remains intact. Implanted diaphragm EMG electrodes (cyan) record impaired (red) and intact (black) diaphragm activity. Intact diaphragm activity exceeding a threshold triggers epidural stimulation to the phrenic motor pool delivered via implanted electrodes (yellow).

To accomplish this, we developed a closed-loop epidural stimulation (CLES) paradigm using endogenous respiratory output to trigger CLES of the cervical spinal cord after cSCI (Fig. 1). Here, rats received a spinal hemisection at C2 (C2HS) to ablate function in the ipsilesional hemidiaphragm. Subsequently, rats received 3 d of chronic, submotor threshold cervical CLES triggered from the functional, contralesional diaphragm EMG. Each day, for 4 d after termination of the electrical stimulation, we measured changes in spinal motor evoked potentials (sMEPs) to test the hypothesis that chronic CLES elicits spinal respiratory neuroplasticity (plasticity is a change in a neural control system based on prior experience; Mitchell and Johnson, 2003) and, therefore, could present a means of restoring independent breathing after cSCI.

## Materials and Methods

### Animals

Experiments were performed on three- to five-month-old female Sprague Dawley rats (Envigo) weighing 250–300 g. Rats were housed one-per-cage during experimentation, provided with food and water *ad libitum*, and maintained under a 12/12 h light/dark cycle. All experimental protocols were performed under the approval and guidance of the Institutional Animal Care and Use Committee at the University of Florida and conformed to policies found within the National Institutes of Health *Guide for the Care and Use of Laboratory Animals*.

### Experimental design and statistical analysis

Female Sprague Dawley rats were split into four groups: (1) rats with a C2HS SCI that received CLES therapy, C2HS + CLES (*n* = 8); (2) rats with a C2HS SCI that did not receive therapy, C2HS (*n* = 6); (3) rats with no SCI that received CLES therapy, intact + CLES (*n* = 6); and (4) rats with no SCI that did not receive therapy, intact (*n* = 6). Rats received a C2HS (or sham surgery) and were instrumented with indwelling bilateral diaphragm EMG recording electrodes and bilateral cervical epidural stimulating electrodes at the C4 spinal level. After 1 to 2 d of postsurgical recovery, sMEP and electrochemical impedance spectroscopy (EIS) were recorded as each animal’s baseline, denoted D0 (Fig. 1*A*). In rats that received stimulation, CLES was then administered 12–20 h/d for three consecutive days, and sMEP and EIS were recorded after the stimulation period on each day (D1–D3). CLES, sMEP, and EIS methods are explained in more detail below. For analysis of sMEPs, mixed effect models were fit to peak-to-peak data. This model was chosen because it is particularly well suited for hierarchically structured data. A variable indicating the comparison of interest (i.e., injury and/or CLES therapy), the log transformation of stimulation amplitude, and the interaction term between these two variables were included in the model. The random effect was defined as the log transformation of stimulation amplitude for each rat. Values of *p *<* *0.05 were considered statistically significant. Additional analyses of sMEPs included motor threshold, peak time, and peak-to-peak amplitude across time bins. Motor threshold is defined as the minimum current sufficient to elicit a discernible sMEP for a given animal on a given day. Differences in motor threshold were determined by Welch’s *t* test. Peak sMEP time is defined as the time (ms) between the stimulus that evokes the sMEP and the peak of the sMEP signal. Differences in peak time were determined via two-way ANOVA with Tukey’s HSD *post hoc* test. Time bins were measured in ms from the onset of the stimulus that evokes the sMEP. Differences in time bins were determined via a mixed effects model. The log transformation of stimulus amplitude, the day of measurement, the time bin, and the interaction term between these variables were included in the model. The random effect was defined as the log transformation of stimulation amplitude for each rat. The R package, report, was used to generate reports for ANOVA, Tukey’s HSD, and Welch’s *t* tests (Makowski et al., 2020). To test for changes in impedance magnitude from day 0 to day 3 within each animal group, impedance magnitude at 1, 10, and 100 kHz was extracted from the spectra. A two-way, repeated measures ANOVA was applied for each of the three magnitudes to determine whether the interaction between day and group was significant on the 1 Hz, 10 kHz, and 100 kHz impedances. The complex impedance data were also visualized using Nyquist plots to further characterize changes in impedance from day 0 to day 3 for each group. All statistical testing was performed in RStudio 1.4.1103 software.

### Electrode fabrication

Epidural stimulating electrodes were made with 10-cm lengths of 40 AWG FEP-insulated stranded stainless-steel wires (Cooner Wire) soldered to stainless-steel pins (P1 Technologies) and set into a 6-pin Delrin electrode pedestal (P1 Technologies) secured with 2 Ton Epoxy (Devcon). The complete stimulating unit consisted of two stimulating wires for epidural implantation and one ground wire. Two stimulating wires had a 2-mm length of insulation shaved off 1 mm from the end and the exposed cut ends were capped with adhesive silicone (MED-1137, NuSil Technology). The ground wire had a 1-cm-long de-insulated end. Diaphragm EMG recording electrodes were made with 20-cm lengths of 36 AWG PFA-insulated seven-stranded stainless-steel wires (AM Systems) soldered to stainless-steel pins and set into a six-pin electrode pedestal as described above. The complete recording unit consisted of four recording wires (two per hemidiaphragm) and one ground wire, which was fabricated as described above.

### Surgery, SCI, and electrode implantation

Rats were anesthetized with 2% isoflurane and given 2 mg/kg of Meloxicam (Patterson Veterinary) and 0.05 mg/kg of buprenorphine (CIII, Patterson Veterinary) preoperatively. Rats were maintained via nose cone and a precision gas vaporizer (1–2% isoflurane). Throughout all surgical procedures, body temperature was maintained at 37–38°C and level of anesthesia was periodically assessed with toe pinch and the palpebral reflex. Rats were given 4-ml subcutaneous injections of lactated ringer’s solution at the start, midpoint, and completion of surgery. After laparotomy, four EMG electrodes were surgically implanted bilaterally (two leads per side) on the diaphragm muscle. A 27-gauge needle bent at a 90° angle was inserted into the superficial layer of diaphragm muscle and recording electrode wires were threaded into the needle and the needle was then withdrawn. A small 3- to 5-mm portion of the wire was de-insulated perioperatively and knots of 6–0 suture were then attached to the wire to secure it in place. After closing, rats were then placed prone, and a laminectomy was performed at C2 and C4. Epidural stimulating wires were threaded under the C5 lamina and sutured to the dura over C4 with 9–0 suture, then at C2 a small incision was made in the dura followed by a C2HS of the left side of the spinal cord (Fig. 1*B*). This injury led to immediate paralysis/paresis of the ipsilesional hemidiaphragm while leaving the contralesional diaphragm functional. C2HS is an established model of upper cSCI and its effects are well characterized (Goshgarian et al., 1986; Fuller et al., 2006, 2008; Vinit et al., 2006; Doperalski et al., 2008; Lane et al., 2009; Sandhu et al., 2009; Lee et al., 2013; Gill et al., 2014). Musculature and skin were closed in layers with 4–0 suture. Wires from epidural stimulating and EMG recording electrodes were threaded subcutaneously to the skull, and both 6-pin electrode pedestals were affixed with stainless steel skull screws and dental acrylic. Drops of lidocaine 2%/epinephrine 1:100,000 were locally administered to prevent excessive bleeding. Rats were housed in incubators for 24–48 h postoperatively at 80°F and 55% humidity, and buprenorphine (0.05 mg/kg; q 8–12 h) and meloxicam (5.0 mg/kg; q 24 h) were administered for 3–5 d.

### Chronic CLES and diaphragm EMG recording

Implanted epidural stimulating electrodes, diaphragm recording electrodes, and a subcutaneous reference electrode were connected to the Neurochip3 brain-computer interface (Jackson et al., 2006b; Zanos et al., 2011; Shupe et al., 2021) via a six-channel cable (P1 Technologies) plugged into the implanted headstage and electrode assembly. The Neurochip3 is a battery-powered, integrated recording, computing, and stimulating system. It was used in this experiment to record diaphragm EMG, digitize the EMG signal, and deliver CLES based on user-defined events within the EMG signal (Fig. 1*B*). Setting relevant recording, event, and stimulation parameters was accomplished through a MATLAB R2020a (The MathWorks) graphical user interface. Diaphragm EMG was recorded at 10 kHz and bandpass filtered (10 Hz to 1 kHz). Stimulation was triggered when the contralesional EMG signal exceeded roughly 80% of its maximum amplitude. When this threshold was crossed, a single pulse (biphasic, symmetric, charge-balanced, cathode leading, 0.2 ms per phase, square wave) was delivered differentially to the epidural stimulating electrodes. This thresholding condition resulted in stimulation being delivered in-phase with diaphragm contraction with trains at a rate of ∼40–100 pulses per second. The amplitude of stimulation was determined uniquely for each rat on each day because of differing sensitivity (i.e., amplitude was kept well below that which could cause pain or discomfort). The use of unique stimulation amplitude that may vary between individuals and across time is a well-known phenomenon in the epidural stimulation field (Atkinson et al., 2011; Angeli et al., 2018; Calvert et al., 2019; Chandrasekaran et al., 2020). Stimulation amplitudes were below motor threshold (defined as the minimum current sufficient to elicit a discernible sMEP and checked each day) for each rat and were typically between 169 and 195 μA (range = [60, 260] μA; mean = 176 μA; mode = 195 μA; 25th percentile = 169 μA; 50th percentile = 188 μA; 75th percentile = 195 μA). This stimulation was typically applied for 12–22 h a day for each day of the experiment (range = [1.5, 28] h; mean = 16 h; mode = 12 h; 25th percentile = 12 h; 50th percentile = 12 h; 75th percentile = 22 h). Stimulation duration was intended to be delivered for as long as possible but varied for several reasons including experiment/facility/personnel time constraints, batteries losing charge, and animal electrode headcaps becoming unplugged. However, this variance was recorded and accounted for. The Collapsed variables section below details how the impact of these variables on the peak-to-peak amplitude outcome was tested. These variables were ultimately collapsed, and the data pooled because they did not have a significant impact on peak-to-peak amplitude. For the sake of naming convention in intact rats which would not have ipsilesional or contralesional hemidiaphragm EMG recordings, “ipsilesional” refers to the left hemidiaphragm, while “contralesional” refers to the right hemidiaphragm.

### sMEPs

The sMEP serves as a useful marker of spinal excitability and is used widely in clinical and experimental settings. Here, this was used to quantify change in the excitability of the phrenic motor system (Bestmann and Krakauer, 2015). sMEPs were measured daily via diaphragm recording electrodes after the day’s CLES period concluded. Diaphragm EMG signals were amplified (Model 1700 Differential AC Amplifier, AM Systems) with a gain of 1000× and bandpass filtered (10 Hz to 5 kHz), then digitized and recorded by the data acquisition device (Power1401-3A, Cambridge Electronic Design Limited). Contralesional EMG signal was processed as a moving-average (MA-821, CWE; time constant 50 ms) before being sent to the data acquisition device. Data were recorded using Spike2 v10 software (Cambridge Electronic Design Limited). Contralesional EMG signals were used to trigger stimulus pulses on inspiration since this is the most effective at eliciting evoked potentials from the diaphragm (Sunshine et al., 2018). When this signal reached 80% of maximum inspiratory amplitude, a single pulse (biphasic, symmetric, charge-balanced, cathode leading, 0.2 ms per phase, square wave) was delivered differentially to the epidural stimulating electrodes at C4 with an interpulse interval of at least 1.5 s to avoid temporal summation. The stimulus intensity was increased stepwise from 100 to 600 μA in 10- or 100-μA increments with 10–20 stimulus pulses at each intensity. The resulting diaphragm EMG activity evoked from each group of the stimuli were averaged to produce stimulus-triggered averages within Spike2 software. Spike2 was then used to extract peak-to-peak amplitude of the stimulus-triggered average, which was measured across 1- to 21-ms windows after the stimulus pulse. Peak-to-peak amplitude was plotted for each rat, day, and stimulus amplitude to generate stimulus-response curves. Stimulus-response curves were generated to assess neural efficacy.

### EIS

Electrochemical characterization of all electrodes was performed via EIS using an Autolab potentiostat/galvanostat (PGSTST128N, Metrohm). Before implantation, each electrode was tested in a two-electrode configuration to mimic *in vivo* conditions and verify that each electrode was functioning as expected. The stimulation electrode served as the working electrode, the large surface area tissue ground wire as the reference and counter electrodes, and phosphate buffered saline as the electrolyte. After implantation, EIS measurements were made using the same two-electrode set-up, with the stimulation electrode serving as the working electrode, and a large surface area stainless-steel electrode as the reference/counter-electrode implanted subcutaneously. EIS measurements were taken by applying a small 20-mV peak-to- peak sinusoidal perturbation to the working electrode. The frequency was swept logarithmically over 20 points from 100 kHz to 10 Hz. The potential difference between the working electrode and the reference electrode was recorded, and the impedance was determined by performing a numerical Fourier analysis. EIS measurements were recorded daily throughout the study to monitor electrode functionality and to test for differences between groups and ipsilesional and contralesional electrodes.

### Software

Software used to complete this study includes Spike2 v10, MATLAB R2020a, Python 3.8.8, and RStudio 1.4.1103. Spike2 was used to interface with the stimulation and recording hardware during sMEP sessions and to deliver stimulus pulses and record EMG. Spike2 was also used to extract relevant sMEP data (e.g., peak-to-peak amplitude) from the EMG recordings. A graphical user interface within MATLAB enabled configuration of the Neurochip3 hardware that controlled CLES. Python was used to aggregate, explore, and clean data. All statistical analysis and plotting were done within RStudio. For more details on statistical analysis, see above, Experimental design and statistical analysis.

### Collapsed variables

Several variables in this study were not strictly controlled because of variability inherent in a study on freely behaving animals. These variables included the duration of CLES, time of day of sMEP measurement, and time between the end of CLES and beginning of sMEP measurement on a given day. These variables were analyzed to determine whether they affected the sMEP peak-to-peak amplitude outcome measure. They had very low correlations with peak-to-peak amplitude and had nonsignificant *p* values and small coefficients in a multiple linear regression with peak-to-peak amplitude as the dependent variable. Thus, these variables were collapsed, and the data pooled.

## Results

### Impact of chronic, closed-loop, cervical epidural stimulation on sMEP peak-to-peak amplitude

**Figure 2.**
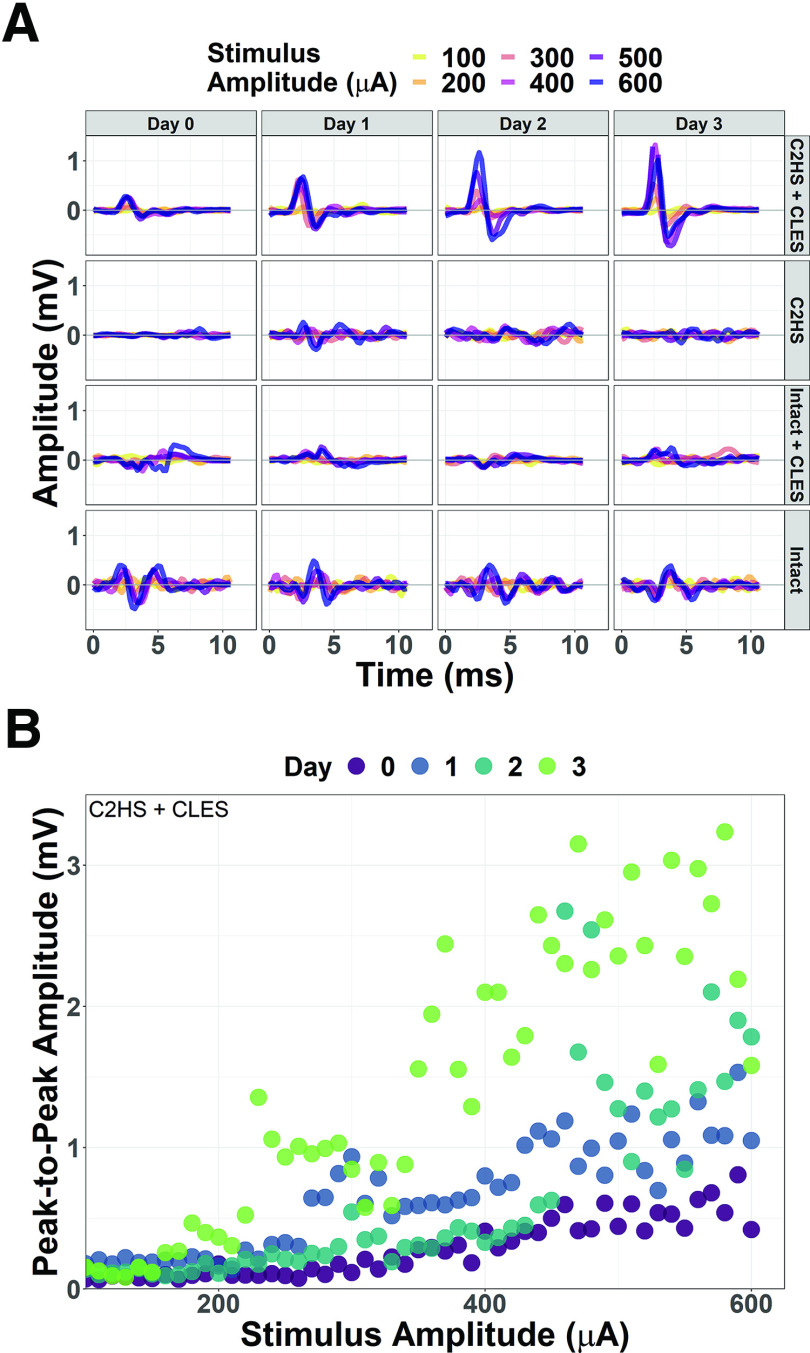
Representative sMEP over the course of 3 d of CLES. ***A***, Representative ipsilesional sMEP from a single rat in each group show that, as expected, larger stimulus amplitudes elicited larger sMEP within a given day. However, sMEP amplitude also notably increased across days for a given amplitude within the C2HS + CLES group, which we interpret as evidence of respiratory neuroplasticity. ***B***, Ipsilesional peak-to-peak amplitudes plotted from the C2HS + CLES group in ***A*** show sMEP peak-to-peak amplitude increases over time.

**Figure 3.**
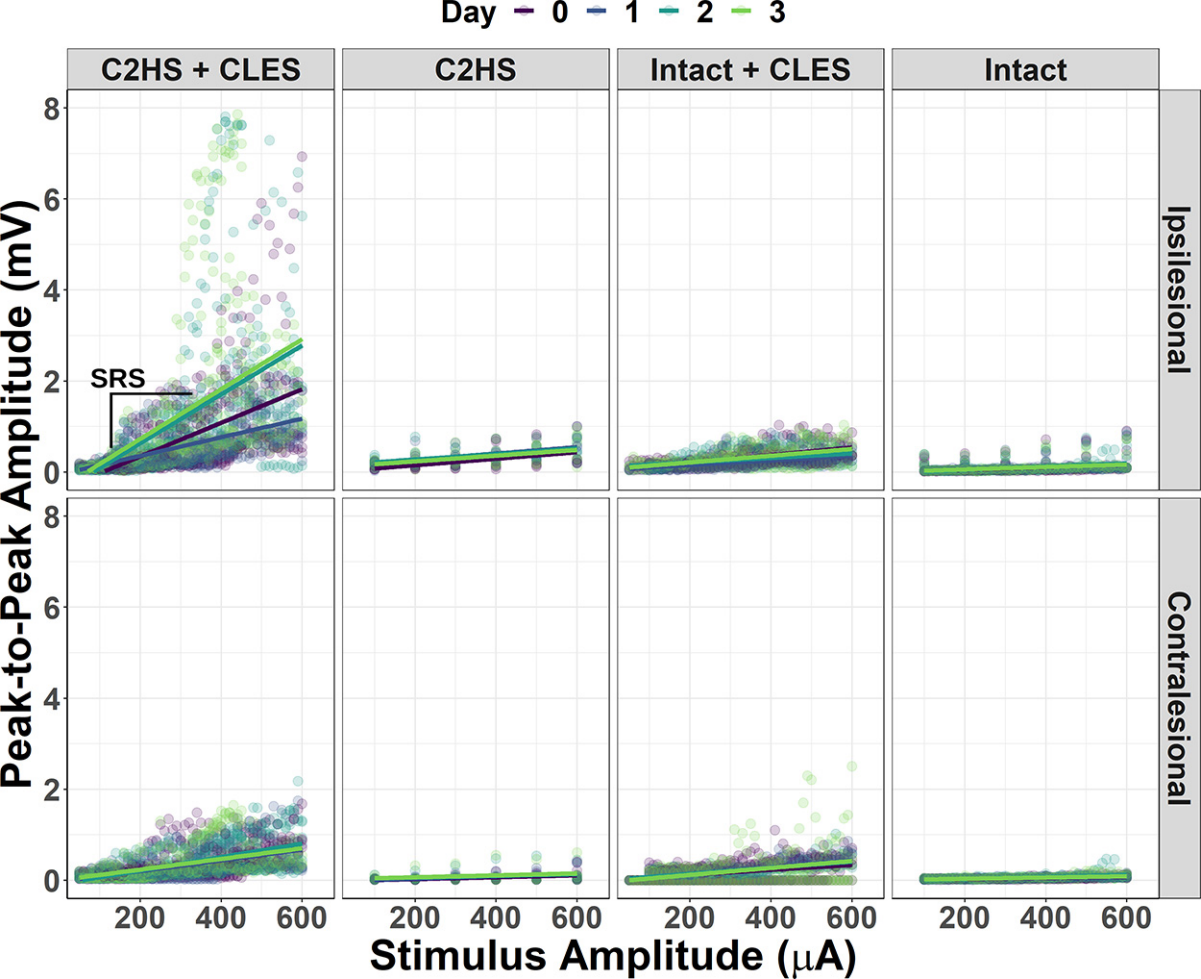
sMEPs, group data. All data points for all animals are plotted along with lines (stimulus-response curves) fit with simple linear regression using ordinary least squares for each group, side, and day. One stimulus-response curve has been annotated to illustrate its stimulus response slope (SRS). Ipsilesional data show an increase in the SRS across days that is most pronounced in C2HS + CLES rats. Contralesional data show smaller increases in the slope of fit stimulus-response curves across days compared with that seen in ipsilesional C2HS + CLES animals. This plot serves to illustrate trends seen in the aggregate data and motivate further statistical analysis. It does not communicate any statistical comparisons.

Representative traces (Fig. 2*A*) and measurements (Fig. 2*B*) of peak-to-peak amplitude of stimulus-triggered averages for single rats show that peak-to-peak amplitude increases with stimulus amplitude, but robust facilitation over the course of the study only occurs for the C2HS + CLES rats. Note that the peak-to- peak amplitude increases over time (e.g., from day 0 to day 3) given the same stimulus amplitude, especially with increasing current. Group data (Fig. 3) show that sMEP peak-to-peak amplitude was facilitated in C2HS + CLES rats indicated by increased stimulus-response slopes (SRSs) for days 1–3 compared with day 0, which was not present in other animal groups.

### Stimulus-response curves: C2HS + CLES versus C2HS alone

**Figure 4.**
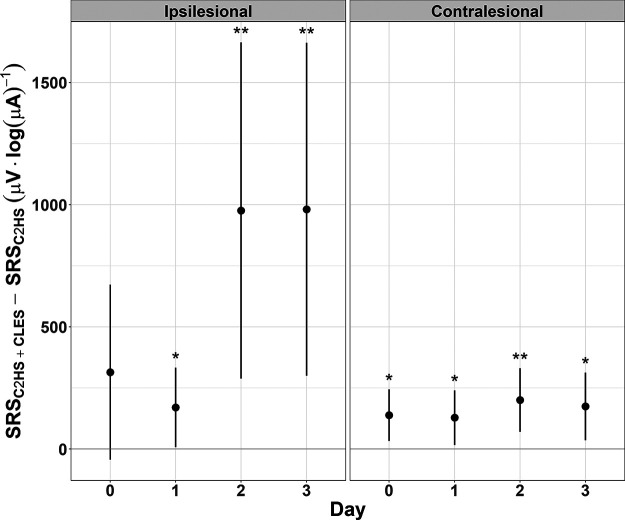
Differences between the SRS of C2HS + CLES rats and C2HS rats. Comparing the difference in ipsilesional SRS shows no difference between C2HS + CLES rats and C2HS rats at day 0. However, each day thereafter, the slope for C2HS + CLES rats is significantly larger. The largest estimates of this difference are on days 2 and 3. Contralesional data show that C2HS + CLES rats have a larger SRS than C2HS rats each day, albeit with smaller difference estimates. The only comparisons tested here are between C2HS + CLES and C2HS rats for a given day and side (linear mixed effects model, *p ≤* *0.05, **p ≤* *0.01; dots indicate the model’s estimate; lines indicate the 95% confidence interval). Positive values here indicate that the C2HS + CLES group had a larger SRS than the C2HS group.

To assess the effect of CLES, we tested the hypothesis that CLES elicits facilitation after C2HS in sMEP peak-to-peak amplitude (vs C2HS alone), linear mixed effects models were fit to each day’s stimulus-response data for each side, ipsilesional and contralesional, to obtain a measure of the difference between SRS of each group, C2HS + CLES and C2HS, for a given day and side (Fig. 4). Of the two groups that received a C2HS, those that also received CLES showed a facilitation in sMEP peak-to-peak amplitude compared with those that did not receive CLES. Furthermore, it is important to note that there was no difference in ipsilesional SRSs between the two groups on day 0, but the ipsilesional SRS was larger in C2HS + CLES rats than in C2HS rats on day 1 (difference estimate = 169.8 μV·log(μA)^−1^, CI [7.1, 332.5], *p *=* *0.041), day 2 (difference estimate = 975.8 μV·log(μA)^−1^, CI [287.9, 1663.8], *p *=* *0.005), and day 3 (difference estimate = 981 μV·log(μA)^−1^, CI [299.4, 1662.6], *p *=* *0.005). The contralesional SRS was larger in C2HS + CLES rats than in C2HS rats on day 0 (difference estimate = 138.4 μV·log(μA)^−1^, CI [32.7, 244.2], *p *=* *0.01), day 1 (difference estimate = 128.1 μV·log(μA)^−1^, CI [16.4, 239.8], *p *=* *0.025), day 2 (difference estimate = 200.2 μV·log(μA)^−1^, CI [69.9, 330.6], *p *=* *0.003), and day 3 (difference estimate = 174.2 μV·log(μA)^−1^, CI [35.6, 312.8], *p *=* *0.014). It is noteworthy that the difference estimates for the contralesional, intact side were smaller than the ipsilesional difference estimates and did not show an increase over time like that seen on the ipsilesional side. Because sMEPs were measured following the conclusion of CLES each day, ipsilesional data suggest that CLES after C2HS elicits respiratory neuroplasticity, and when contralesional data are considered, we see that an injury may be necessary to enable CLES-induced facilitation in SRS over time.

### Stimulus-response curves: C2HS + CLES versus intact + CLES

**Figure 5.**
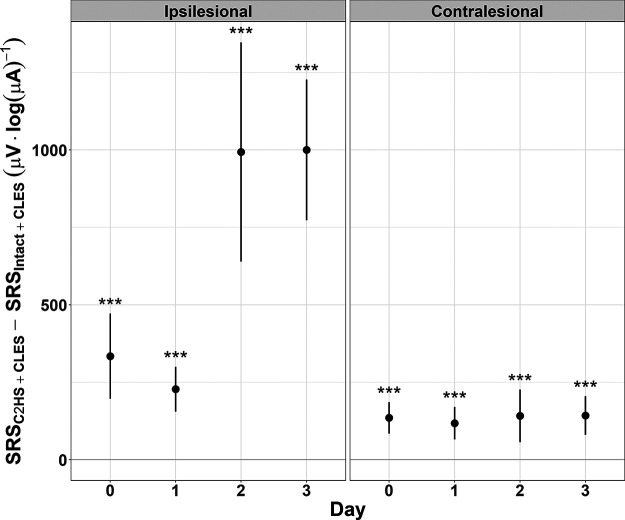
Differences between the SRSs of C2HS + CLES rats and intact + CLES rats. Comparing the difference in ipsilesional SRS shows that on each day, the SRS for C2HS + CLES rats is significantly larger than the SRS for intact + CLES rats, and the largest estimates occur on day 2 and day 3. Contralesional data show that C2HS + CLES rats have a larger SRS than C2HS rats each day, albeit with smaller difference estimates. The only comparisons tested here are between C2HS + CLES and intact + CLES rats for a given day and side (***p ≤* *0.001; dots indicate the model’s estimate; lines indicate the 95% confidence interval). Positive values here indicate that the C2HS + CLES group had a larger SRS than the intact + CLES group.

To assess the effect of the hemisection injury, we tested the hypothesis that CLES elicits a more robust facilitation of sMEP peak-to-peak amplitude in C2HS versus intact rats. Linear mixed effects models were fit to each day’s stimulus-response data for each side, ipsilesional and contralesional, to obtain a measure of the difference between the SRS of each group, C2HS + CLES and intact + CLES, for a given day and side (Fig. 5). Of the two groups that received CLES, those that also had a C2HS injury showed a facilitation in sMEP compared with those that did not have a C2HS injury (intact). Ipsilesional and contralesional SRSs were larger every day in C2HS + CLES rats versus in intact + CLES rats. However, the ipsilesional side showed larger difference estimates that also increased over time. Specifically, the ipsilesional SRS was larger in C2HS + CLES rats than in intact + CLES rats on day 0 (difference estimate = 334.2 μV·log(μA)^−1^, CI [196.6, 471.8], *p *<* *0.001), day 1 (difference estimate = 227.7 μV·log(μA)^−1^, CI [155.3, 300.2], *p *<* *0.001), day 2 (difference estimate = 993.4 μV·log(μA)^−1^, CI [639.8, 1346.9], *p *<* *0.001), and day 3 (difference estimate = 1000.1 μV·log(μA)^−1^, CI [773, 1227.3], *p *<* *0.001). The contralesional SRS was larger in C2HS + CLES rats than in intact + CLES rats on day 0 (difference estimate = 135 μV·log(μA)^−1^, CI [84.4, 185.7], *p *<* *0.001), day 1 (difference estimate = 117.7 μV·log(μA)^−1^, CI [65.1, 170.4], *p *< 0.001), day 2 (difference estimate = 141.5 μV·log(μA)^−1^, CI [56.3, 226.7], *p *=* *0.013), and day 3 (difference estimate = 142.6 μV·log(μA)^−1^, CI [80.2, 205], *p *<* *0.001). In summary, the ipsilesional SRS was always significantly larger in C2HS + CLES animals than in intact + CLES animals, and the difference estimates increased over time. The contralesional SRS was also always larger in C2HS + CLES animals than in intact + CLES animals, but the difference estimates did not show the same marked increase over time. Together, these results suggest that CLES does not cause the same degree of sMEP facilitation in intact rats as it does in rats with a C2HS. Thus, C2HS may allow for apparent marked facilitation.

### Stimulus-response curves: ipsilesional versus contralesional

**Figure 6.**
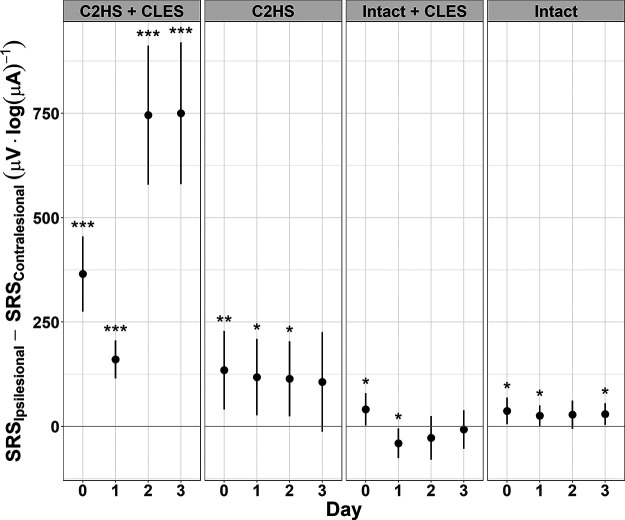
Differences between the ipsilesional and contralesional SRSs for each rat group. For C2HS + CLES rats, the ipsilesional SRS is larger than the contralesional SRS each day, and the largest differences occur on days 2 and 3. For C2HS rats, ipsilesional SRS is larger than the contralesional SRS on days 0, 1, and 2. There is no difference on day 3. For intact + CLES rats, ipsilesional SRSs are larger on day 0 and smaller on day 1 than the contralesional SRS. There is no difference on day 2 or 3. For intact rats, the ipsilesional SRS is larger than the contralesional SRS on days 0, 1, and 3. There is no difference on day 2. It is noteworthy that the only group that shows ipsilesional SRSs that are larger than contralesional SRSs each day is the C2HS + CLES group. The only comparisons tested here are between the ipsilesional and contralesional side for a given group on a given day (*p ≤* *0.05, **p ≤* *0.01, ***p ≤* *0.001; dots indicate the model’s estimate; lines indicate the 95% confidence interval). Positive values here indicate that the ipsilesional side had a larger SRS than the contralesional side.

To assess the unilaterality of the effect of CLES, we tested the hypothesis that ipsilesional sMEPs show greater facilitation compared with contralesional sMEPs within C2HS + CLES rats. Linear mixed effects models were fit to each day’s stimulus- response data for each rat group to obtain a measure of the differences between the SRS of each side, ipsilesional and contralesional, for a given day and group (Fig. 6). Ipsilesional SRS was larger every day in C2HS + CLES and this result was absent in other rat groups: C2HS, intact + CLES, intact. Specifically, the SRS for C2HS + CLES rats was larger on the ipsilesional side than on the contralesional side on day 0 (difference estimate = 365.1 μV·log(μA)^−1^, CI [275.2, 454.9], *p *<* *0.001), day 1 (difference estimate = 160.4 μV·log(μA)^−1^, CI [115, 205.7], *p *<* *0.001), day 2 (difference estimate = 745.6 μV·log(μA)^−1^, CI [578.9, 912.4], *p *<* *0.001), and day 3 (difference estimate = 750 μV·log(μA)^−1^, CI [580.3, 919.6], *p *<* *0.001). However, C2HS alone does not facilitate SRSs over 3 d. Specifically, the SRS for C2HS rats was larger on the ipsilesional side than on the contralesional side on day 0 (difference estimate = 134.5 μV·log(μA)^−1^, CI [40.4, 228.7], *p *=* *0.005), day 1 (difference estimate = 118 μV·log(μA)^−1^, CI [26.4, 209.6], *p *=* *0.012), and day 2 (difference estimate = 113.9 μV·log(μA)^−1^, CI [23.9, 203.8], *p *=* *0.013), but disappears on day 3. This initial facilitation in ipsilesional versus contralesional sMEP stimulus-response curves for C2HS rats may suggest hyperexcitability after C2HS. It is important to note that these sMEP SRS estimates for C2HS rats are not as large as those for C2HS + CLES rats, especially on days 2 and 3. In intact + CLES rats, we see no persistent facilitation across time, and any significant difference estimates are relatively small in magnitude. Specifically, the SRS for intact + CLES rats was larger on the ipsilesional side than on the contralesional side on day 0 (difference estimate = 40.6 μV·log(μA)^−1^, CI [1.9, 79.4], *p *=* *0.04); was smaller on the ipsilesional side than on the contralesional side on day 1 (difference estimate = −40.6 μV·log(μA)^−1^, CI [−76.1, −5], *p = *0.025) and was not different between the ipsilesional and contralesional side on days 2 and 3. Lastly, intact animals showed some difference between ipsilesional and contralesional SRS, but these estimates are small in magnitude and did not increase over time. Specifically, the SRS for intact rats was larger on the ipsilesional side than on the contralesional side on day 0 (difference estimate = 37 μV·log(μA)^−1^, CI [5, 69], *p *=* *0.023) and on day 1 (difference estimate = 25.4 μV·log(μA)^−1^, CI [0.4, 50.5], *p *=* *0.047); was not different between the ipsilesional and contralesional side on day 2; and was larger on the ipsilesional side than on the contralesional side on day 3 (difference estimate = 29.4 μV·log(μA)^−1^, CI [3.2, 55.6], *p *=* *0.028). In summary, these models indicate that C2HS alone caused some initial facilitation, which disappeared by day 3, and when paired with CLES, the facilitation markedly increases over time.

### Stimulus-response curves: difference from day 0

**Figure 7.**
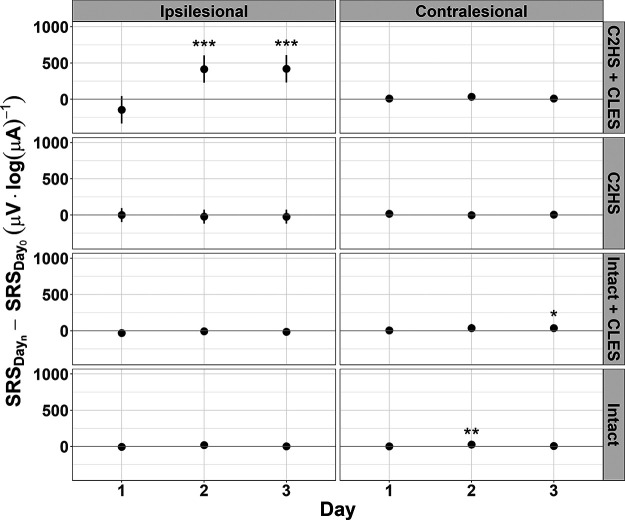
Differences between the SRSs for each rat group and each side. For every rat group and each side, there is never a significant difference between the day 1 SRS and the day 0 SRS. However, for the ipsilesional side of C2HS + CLES rats, the day 2 and day 3 SRSs are larger than the day 0 SRS. The only other significant differences are for contralesional side of intact + CLES rats on day 3 and the contralesional side of intact rats on day 2. However, these differences are noticeably smaller than the differences seen for ipsilesional C2HS + CLES SRSs. The only comparisons tested here are between a given day and day 0 for a given side and rat group (*p ≤* *0.05, **p ≤* *0.01, ***p ≤* *0.001; dots indicate the model’s estimate; lines indicate the 95% confidence interval). Positive values here indicate that the given day had a larger SRS than day 0.

To assess the temporal effect of the CLES, we tested the hypothesis that sMEP peak-to-peak amplitude facilitated in C2HS + CLES rats on days 1, 2, and 3 versus day 0. Linear mixed effects models were fit to each side’s stimulus-response data for each rat group to obtain a measure of the differences between the SRS on day 0 and each subsequent day (days 1–3), for a given side and group (Fig. 7). We found that only in the ipsilesional SRS of the C2HS + CLES group is facilitation present from day 0 to subsequent days. Specifically, the ipsilesional SRS for C2HS + CLES rats was not different from day 0 on day 1; was larger than day 0 on day 2 (difference estimate = 415.8 μV·log(μA)^−1^, CI [228.8, 602.9], *p *<* *0.001); and was larger than day 0 on day 3 (difference estimate = 419.4 μV·log(μA)^−1^, CI [230.8, 607.9], *p *<* *0.001). The contralesional SRS for C2HS + CLES rats was not different from day 0 on day 1, 2, or 3. For C2HS rats, both the ipsilesional SRS and contralesional SRS were not different from day 0 on day 1, 2, or 3. For intact + CLES rats, the ipsilesional SRS was not different from day 0 on day 1, 2, or 3. The contralesional SRS for intact + CLES rats was not different from 0 on day 1; was not different from 0 on day 2; and was larger than 0 on day 3 (difference estimate = 37.1 μV·log(μA)^−1^, CI [2.9, 71.3], *p = *0.034). For intact rats, the ipsilesional SRS was not different from day 0 on day 1, 2, or 3. Interestingly, the contralesional SRS for intact rats was larger than day 0 on day 2 (difference estimate = 26.9 μV·log(μA)^−1^, CI [11.3, 42.4], *p = *0.001) although not different from 0 on day 1 on day 3. This effect, however, was small. Collectively, these data suggest that CLES elicits respiratory neuroplasticity after C2HS because there is a persistent change in the SRS over time in C2HS + CLES rats on the ipsilesional side. No other groups show this persistent facilitation. Additionally, note that any significant difference estimates not in ipsilesional C2HS + CLES rats were of such low magnitude that they may be considered negligible.

### Motor threshold

**Figure 8.**
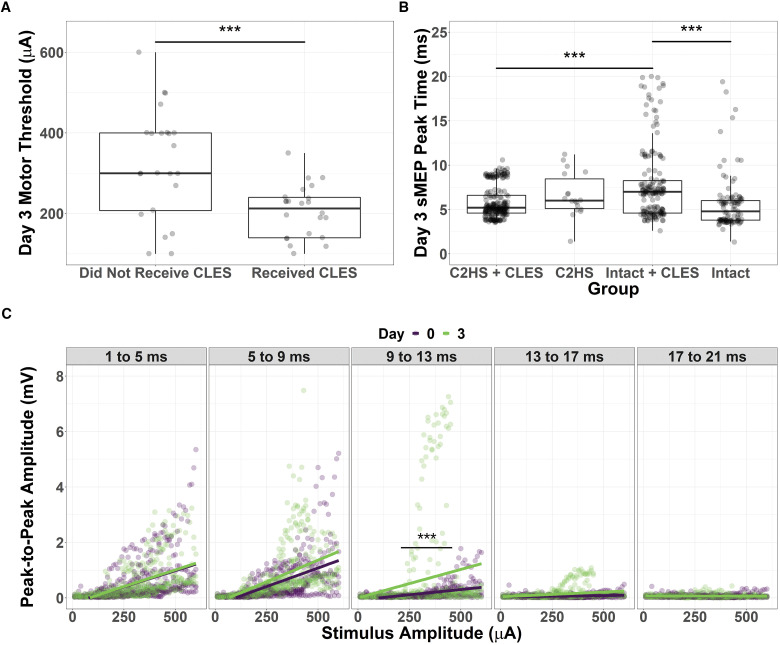
Additional analyses of sMEP. ***A***, Day 3 motor threshold is significantly lower in rats that received CLES than in rats that did not receive CLES (***p ≤* *0.01). The presence of C2HS had no effect. ***B***, The peak time of sMEP on day 3 differed significantly across groups. Intact + CLES rats had larger time-to-peak amplitude values than intact rats and C2HS + CLES rats (***p ≤* *0.001). ***C***, In C2HS + CLES rats, ipsilesional peak-to-peak amplitude was significantly larger in the 9- to 13-ms time bins on day 3 compared with day 0 (***p ≤* *0.001). Data in ***A*** and ***B*** presented as box and whisker plots displaying the 25th, 50th, and 75th percentile. Whiskers extend to the maximum and minimum data values that lie within 1.5 times the interquartile range from the hinges.

On day 3 of the experiment, the amount of current necessary to evoke an sMEP was significantly lower in rats that received CLES compared with rats that did not receive CLES (Fig. 8*A*). The Welch two-sample *t* test testing the difference of motor threshold by therapy (mean in group No = 317.08 μA, mean in group Yes = 198.40 μA) suggests that the effect is negative, statistically significant, and large (difference = −118.68 μA, 95% CI [54.61, 182.76], *t*_(33.28)_ = 3.77, *p *<* *0.001; Cohen’s *d* = 1.31, 95% CI [0.55, 2.05]). There was no difference between the ipsilesional and contralesional side. The presence or absence of C2HS did not significantly affect motor threshold. These results suggest that 3 d of CLES to the cervical spinal cord increases the excitability of the phrenic motor network.

### Time-to-peak amplitude

The sMEP peak time in the intact + CLES group was significantly higher than in the C2HS + CLES group (2.04 ms, CI [1.25, 2.83], *p *<* *0.001) and the intact group (2.26 ms, CI [1.29, 3.22], *p *<* *0.001; Fig. 8*B*). The two-way ANOVA suggests that: the main effect of Therapy is statistically significant (*F*_(1,518)_ = 8.85, *p* = 0.003; Eta2 (partial) = 0.02, 90% CI [3.36e-03, 0.04]); the main effect of injury is statistically significant (*F*_(1,518)_ = 32.66, *p* < 0.001; Eta2 (partial) = 0.06, 90% CI [0.03, 0.10]); and the interaction between Therapy and injury is statistically significant (*F*_(1,518)_ = 13.85, *p* < 0.001; Eta2 (partial) = 0.03, 90% CI [8.16e-03, 0.05]). CLES may show differential effects on the injured versus intact phrenic motor system, which further supports that C2HS itself may play a role in the ability to detect CLES-induced facilitation

### Peak-to-peak amplitude across time bins

The SRS of the ipsilesional peak-to-peak amplitude was significantly larger on day 3 versus on day 0 in the C2HS + CLES animals in the 9- to 13-ms time bin (difference estimate = 3.06 μV·log(μA)^−1^, CI [1.54, 4.57], *p *< 0.001; Fig. 8*C*). This difference is larger at larger stimulus amplitudes demonstrating that after C2HS and CLES, peak-to-peak amplitude is increased in longer latency time domains. These results suggest that CLES may enable the recruitment of polysynaptic pathways on day 3 and further investigation is warranted. In addition, the effect was much more pronounced on the ipsilesional side suggesting that C2HS may be necessary for CLES to elicit these effects.

### EIS

**Figure 9.**
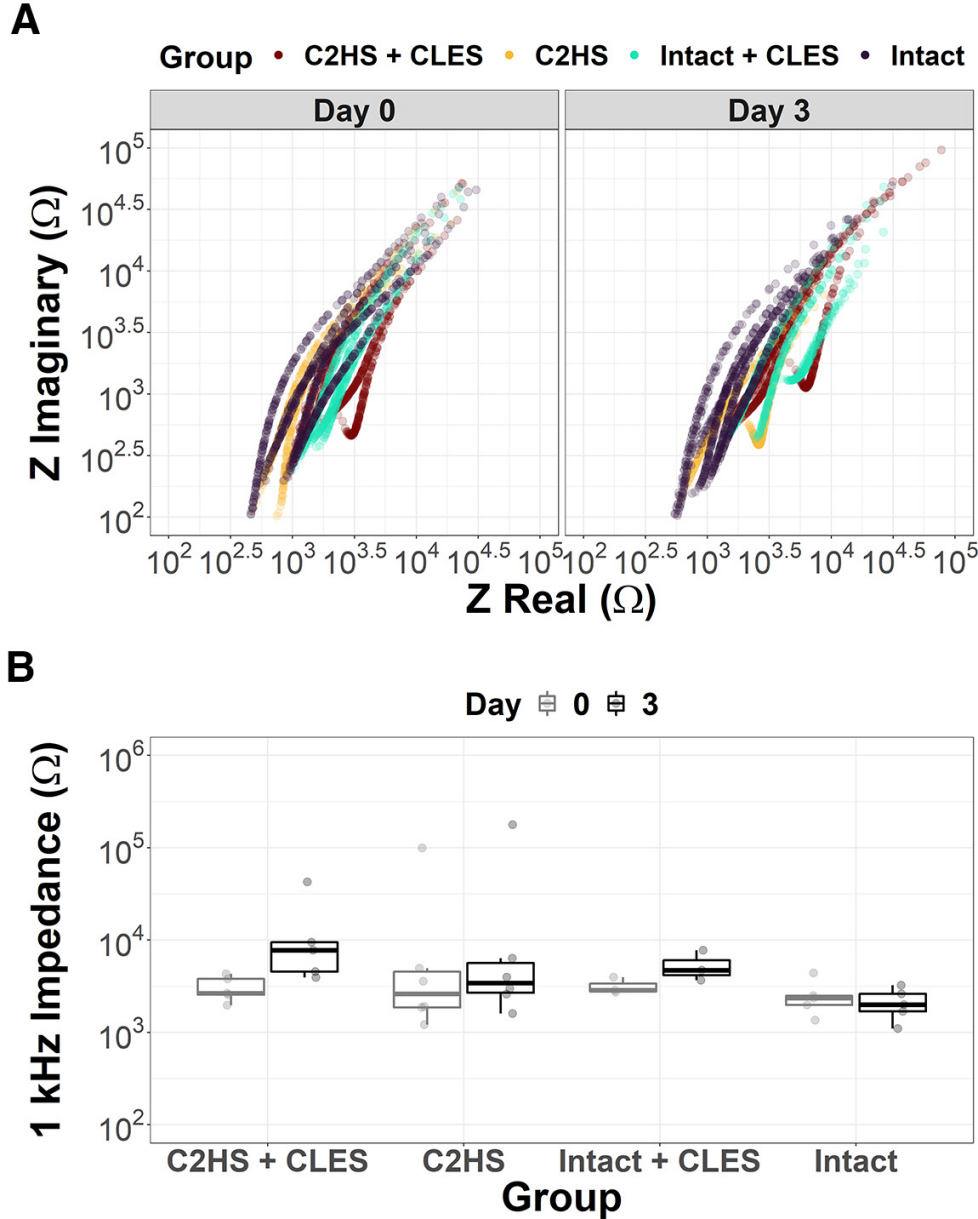
Day 0 and day 3 impedance data from epidural stimulating electrodes. ***A***, Nyquist plots show imaginary impedance (Z imaginary) versus real impedance (Z real). This reveals what appears to be the beginnings of a semi-circular arc at high frequencies, indicative of a possible foreign body response. ***B***, No significant differences in impedance magnitude between groups or days were found suggesting that changes in sMEP reported here are not because of changing physics of the electrode and that any potential electrode encapsulation did not affect the impedance. Data in ***B*** presented as box and whisker plots displaying the 25th, 50th, and 75th percentile. Whiskers extend to the maximum and minimum data values that lie within 1.5 times the interquartile range from the hinges.

No significant differences in impedance magnitude were found between groups or days (Fig. 9). The main effects of day (*F*_(1,30)_ = 0.45, *p* = 0.506; Eta2 (partial) = 0.01, 90% CI [0.00, 0.15]) and group (*F*_(3,30)_ = 1.07, *p* = 0.376; Eta2 (partial) = 0.10, 90% CI [0.00, 0.23]) are not statistically significant. The interaction between day and group is not significant (*F*_(3, 30)_ = 0.10, *p* = 0.960; Eta2 (partial) = 9.78e-03, 90% CI [0.00, 0.00]). No differences were found at 10 nor 100 kHz (data not shown). This validates that the facilitation observed in sMEPs is not because of changing physics of the electrode nor because of biological encapsulation. The formation of a semi-circular arc at high frequencies in the Nyquist space (Fig. 9*A*) is indicative of a possible foreign body response, which could be more impactful at longer timepoints (Williams et al., 2007; Wilks et al., 2012).

## Discussion

To our knowledge, this is the first report that CLES elicits spinal respiratory neuroplasticity after C2HS in freely behaving rats with cSCI. Our findings demonstrate that CLES administered to the region of the phrenic motor nucleus enhances evoked diaphragm motor output and that the magnitude of this enhancement increases across multiple days of therapeutic stimulation. Interestingly, this effect was specific to the ipsilesional diaphragm in C2HS-injured rats (i.e., the effect was not as robust in the contralesional diaphragm). Furthermore, although we observed a small and variable increase in amplitudes of sMEP after CLES in intact rats, it was negligible, suggesting that the injury itself may increase chances for plasticity. In addition, CLES reduced motor thresholds and features appeared in longer-latency time domains of the sMEPS, indicating possible recruitment of synapses. Although mechanisms underlying these changes are unknown and untested, we postulate that these effects could be because of activity-dependent synaptic plasticity. These results are exciting and suggest a potential new route of exploration for use of epidural stimulation as a respiratory therapeutic after cSCI.

### Enabling activation of the phrenic motor network with subthreshold epidural stimulation

Many available therapies, such as functional electrical stimulation (FES), activate respiratory muscles per se versus the neural network controlling them. Unfortunately, direct muscle stimulation produces muscle fatigue, whereas spinal stimulation can enable fatigue-resistant contractions in a more “natural,” coordinated fashion (Moritz, 2018). Thus, many of the reported epidural stimulation paradigms are administered below motor threshold. Chronic, severe SCI greatly reduces the baseline level of spinal network activity, because of the interruption of crucial supraspinal descending inputs (Harkema et al., 2011; Angeli et al., 2014; Gerasimenko et al., 2015; Grahn et al., 2017). Subthreshold epidural stimulation has been hypothesized to greatly improve chances of sensorimotor volitional control by increasing local excitability nearer to motor threshold. Stimulation at current intensities below that which trigger motor activation interacts with ongoing network activity (Ozen et al., 2010) and likely in a way that synchronizes activity of the individual neurons comprising the circuit (Francis et al., 2003; Selverston et al., 2009). Although weak fields do not initiate action potentials (an all-or-none response), they may generate a graded modulation of neurotransmitter release (Mendelson, 1971) thereby potentially increasing resting membrane potentials. Subthreshold stimulation may act similarly on interneuronal or propriospinal networks (Radman et al., 2009). Thus, by initiating a spectrum of subthreshold depolarizations, sensory and propriospinal sources can also be integrated for the fine-tuned modulation required for breathing. Here, we show that the motor threshold for all rats treated with CLES decreased compared with those that did not receive stimulation, indicating components within the phrenic motor network had been potentially augmented from low responsive or nonresponsive to a level whereby evoked potentials could be initiated more easily. In addition, this elevated excitability appeared to enable the appearance of so-called “late responses” (Gerasimenko et al., 2006) characterized by features of the evoked response appearing in later time bins. Late responses are likely to represent polysynaptic events elicited through, in our case, phrenic-related interneuronal populations (Lane et al., 2008). Here, we found that higher amplitude peaks appeared in longer-latency time bins (9–13 ms) after 3 d of stimulation in injured rats that received CLES (although only in the ipsilesional recordings). We hypothesize that features appearing in later time bins may be evidence of this polysynaptic response (Monfils and Teskey, 2004) in respiratory motor circuits. Interneuronal (Lane et al., 2009; Sandhu et al., 2009; Lane, 2011) pathways near the phrenic motor nucleus have been suggested to play an important role in respiratory neuroplasticity after SCI. Additionally, these interneurons may modulate the activity of respiratory motoneurons normally or during conditions of altered respiratory drive (Kirkwood et al., 1993; Streeter et al., 2017, 2020).

### Potential mechanisms of CLES-induced enhancement of evoked responses

Neuroplasticity is a fundamental property of the neural system controlling breathing (Mitchell and Johnson, 2003). We present a form of CLES-induced facilitation of evoked potential magnitude demonstrating at least short-term plasticity since 1) sMEP were recorded after the therapeutic stimulation was turned off for ∼60–120 min, and 2) sMEP magnitude progressively increased over several days in C2HS rats treated with CLES. However, the mechanisms giving rise to CLES-induced enhancement of diaphragm sMEPs are unknown. We do know that the spinal cord retains its ability to learn motor behaviors after SCI via activity-dependent mechanisms (Forssberg et al., 1975; Hodgson et al., 1994; Edgerton et al., 2004; Gad et al., 2013). Restoration of motor function via epidural stimulation in individuals with SCI may partially rely on activity-dependent plasticity of spinal and supraspinal networks (Taccola et al., 2018). Emerging evidence suggests that long-term, closed-loop stimulation in other neural systems may enable lasting neurorehabilitation via activity-dependent processes (Jackson et al., 2006a; Jackson and Zimmermann, 2012; Nishimura et al., 2013). Mechanisms have been postulated as relying on presynaptic and postsynaptic functional changes (Bliss and Collingridge, 2013), monoaminergic modulation (Mauelshagen et al., 1998), and new synapse formation all within the neuromotor network of interest because of the activation of sensory afferents specific to the task (Lavrov et al., 2008; Rejc et al., 2015). In this case, phrenic afferents include large- diameter myelinated fibers (Groups Ia, Ib, and II) that discharge in phase with contraction of the diaphragm (Nair et al., 2017) as well as smaller-diameter, myelinated free nerve endings (Group III) and unmyelinated C fibers (Group IV; Nair et al., 2017). Modeling studies report that epidural stimulation is most likely activating large-diameter, myelinated A-fibers which presumably increases excitability to allow the combination of afferent feedback and any residual descending input to facilitate diaphragm motor output (Taccola et al., 2018). Electrical stimulation of the nervous system also leads to intracellular calcium influx, thereby initiating an ERK-dependent increase in BDNF expression (Wenjin et al., 2011) in the spinal cord (Huie et al., 2012). Indeed, in paradigms of stimulation-induced spinal learning, effects were dependent on the pattern of stimulation, NMDA receptor activation, protein synthesis, and BDNF release (Baumbauer et al., 2009). Collectively, these reports suggest that patterned electrical stimulation of the cervical spinal cord could potentially drive task-specific neuronal activity between residual bulbospinal inputs, afferent sensory inputs, phrenic interneurons and phrenic motoneurons. Thus, delivering stimulation in phase with endogenous respiration may increase the strength and number of terminals in the phrenic motor nucleus via spike-timing-dependent-like plasticity (Nishimura et al., 2013; McPherson et al., 2015; Ganzer et al., 2018). Although we have not tested these hypotheses here, we speculate that CLES-induced respiratory neuroplasticity may be induced via similar mechanisms.

### Epidural electrode placement

This proof-of-principle study marks the first exploration of epidural stimulation to restore respiratory function after an upper cSCI in freely behaving rats. Because these were the first studies of their kind, our rationale to implant the two stimulating electrodes bilaterally over the C4 spinal segment was based largely on anatomy and published reports of phrenic motor circuitry and plasticity (for review, see Malone et al., 2021). Modeling (Struijk et al., 1993; Murg et al., 2000; Capogrosso et al., 2013; Minassian et al., 2016) and cadaver (Swiontek et al., 1976) studies suggest that current is unlikely to spread throughout many segments of the cord. Although the motor neurons within the phrenic motor nucleus typically span from spinal segments C3 to C5, the majority (∼70%) lie within the C4 spinal segment (Goshgarian and Rafols, 1981; Gottschall, 1981). We postulate that CLES is not necessarily directly activating motor neurons per se (e.g., epidural stimulation is hypothesized to activate large diameter afferent fibers first); the cervical dorsal roots carrying diaphragmatic afferents insert at C3-C6 with ∼68% of soma lying within the region of the C4 segment. Subsequent modulation from dorsal inputs to ventral outputs would then occur where the largest proportion of this motor pool resides (C4). Another crucially important C4 neuronal population would include prephrenic interneurons (Lane et al., 2008, 2009; Lane, 2011) which, if activated, could aid in elevating network excitability and lead to respiratory motor recovery (Zholudeva et al., 2018). And finally, crossed latent pathways caudal to the C2 injury (i.e., the crossed phrenic pathway; Goshgarian, 2003, 2009) prompted us to consider a bilateral rather than unilateral placement since the potential for eliciting respiratory plasticity is high in this descending, decussating bulbospinal input to phrenic motor neurons. Other substrates on with CLES could act to elicit plasticity in the C4 region include the dorsal root ganglia (Van Buyten et al., 2015) and glia (Tawfik et al., 2010). In addition, propriospinal neurons at C4 could also allow for recruitment of segmental circuits known to play a role in recovery of respiration after SCI (Jensen et al., 2019). Importantly, low-resistivity cerebrospinal fluid allows for current to flow around the spinal cord (Baumann et al., 1997), suggesting that CLES could also be directly modulating efferent outflow.

Here, we show that CLES-induced facilitation in ipsilesional sMEPs is a much more robust phenomenon than in contralesional diaphragm. While the contralesional phrenic-to-diaphragm network is functional in this model of SCI, it is not correct to state that it is “normal.” In fact, both compensatory plasticity to maintain appropriate ventilation (Lee and Hsu, 2017) as well as diminished responses (vs ipsilesional; Lee et al., 2015) to therapeutics have been reported. We also see a bilaterally attenuated motor threshold and recruitment of polysynaptic pathways ipsilesionally after CLES. Collectively, these data indicate a complex story in which the roles of spinal substrates, latent crossed pathways, as well as cellular mechanistic underpinnings of ipsilesional versus contralesional networks after injury must be investigated to truly advance to a therapeutic outcome.

### Limitations

While we demonstrate that CLES can elicit sMEP plasticity after C2HS, more evidence is needed to assert CLES as a rehabilitative respiratory therapy. Testing the rehabilitative potential of CLES will likely require both longer timepoints postinjury and ultimately need to be repeated and validated in rats with chronic SCI. Here, we have hypothesized that it is the closed-loop nature of the paradigm itself that is beneficial in restoring respiratory function after cSCI for the mechanistic reasons outlined above. Many forms of activity-dependent plasticity require precise timing relationships between presynaptic and postsynaptic activation. Thus, spinal respiratory circuits may be especially responsive and amenable to this approach since pattern sensitivity is a fundamental property of spinal respiratory motor plasticity. However, we have not directly tested this hypothesis here and further work using open-loop or out-of-phase stimulation parameters is required.

Lastly, MEPs serve as a marker of spinal excitability and are used clinically to assess sensorimotor function after SCI. Our current results show enhancement of sMEPs after CLES indicating robust respiratory neuroplasticity. However, we acknowledge that while sMEPS can give insight into the physiology of presynaptic and postsynaptic neural circuits, it is important to link the electrophysiological outcomes to behavior (e.g., breathing recovery). We postulate that an enhancement of sMEP peak to peak amplitude translates to increased synaptic strength with the potential for respiratory recovery. Indeed, enhanced evoked potential amplitude after exposure to other known respiratory neuroplasticity-inducing stimuli (e.g., intermittent hypoxia, cervical spinal A2a receptor agonism) is correlated with increased respiratory motor output (Fuller et al., 2003; Golder et al., 2008; Perim et al., 2021) and ventilation (Golder et al., 2008) in rats. Future experiments are required to understand how CLES affects recovery of breathing after cSCI, but we posit that this is the first step toward developing CLES as a true rehabilitative therapy in this space.

In conclusion, here we demonstrate that CLES induces at least short-term plasticity in sMEP peak-to-peak amplitude after C2HS. To our knowledge, this is the first exploration of CLES-induced plasticity in the respiratory motor control system in freely behaving rats. This study establishes the potential of CLES to promote enduring functional recovery of breathing ability. Although mechanisms of this form of plasticity are unknown, CLES shows great potential as a neuromodulatory strategy to restore breathing function in individuals with severe cSCI, potentially improving the quality of life for thousands of people dependent on mechanical ventilation for survival.

## Synthesis

Reviewing Editor: Jeffery Twiss, University of South Carolina Decisions are customarily a result of the Reviewing Editor and the peer reviewers coming together and discussing their recommendations until a consensus is reached. When revisions are invited, a fact-based synthesis statement explaining their decision and outlining what is needed to prepare a revision will be listed below. The following reviewer(s) agreed to reveal their identity: Angelo Lepore. Note: If this manuscript was transferred from JNeurosci and a decision was made to accept the manuscript without peer review, a brief statement to this effect will instead be what is listed below.

Reviewers noted that you report a novel approach for neuromodulation of the respiratory network for functional recovery with use of closed-loop epidermal stimulation in a rat model of severe cervical spinal cord injury-induced hemi-diaphragm paralysis. Consequently, they felt that development of a system for epidural stimulation of the cervical spinal cord could be a highly valuable approach for the treatment of diaphragmatic respiratory dysfunction after spinal cord injury. The reviewers also raised a number of issues with the manuscript in its present form. Addressing these would strengthen the manuscript and clarify the approach and findings for the readers. In our online discussion, we thought that most of these issues (perhaps all) could be addressed by text edits and additions rather than requiring new experiments. Also, the possibility of having data in hand now that was not included in the submission was also raised. Reviewers did note a lack of mechanistic exploration for a proof-of-principle study, and questioned significance of advance for the field. I fully agree with this point and you should carefully consider this in your revisions.

Major issues:

1.The study does not provide a comprehensive exploration of the approach and importantly does not demonstrate it is superior to the current open-loop stimulation approaches being explored by others. One reviewer expressed concern is that you have not shown that closed-loop stimulation is superior to open-looped stimulation as you predict. However in online discussion, we noted that you may be able to adequately address this in your discussion rather than necessarily insisting on new experiments. I suggest that you emphasize the limitations of the data presented and raise possibility for future studies to directly compare the two. Alternatively, if you have solid data comparing closed and open loop systems, you may want to consider including these.

2.Since the contralateral hemi-diaphragm is functionally intact after the C2 hemisection SCI, why did the authors not restrict the implantation of the epidural stimulating electrodes to only the phrenic nucleus ipsilateral to the injury while still using contralateral hemi-diaphragm activity as the trigger? They present differences in the SRS results between the ipsilesional and contralesional muscle sides, providing interesting data that suggest a potentially selective impact of the injured circuit on the effects of their closed-loop epidural stimulation paradigm. Therefore, there may be some experimental advantages to this bilateral stimulation design; this sided difference in SRS findings was just one example. What about translational considerations for the eventual implantation of such an approach? In general, a discussion of your rationale for the design of stimulation location(s) would be helpful.

3.At an anatomical/circuit level, do the authors believe the epidural stimulation is acting upon the PhMNs directly and/or indirectly on the PhMNs (for example, by activating pre-phrenic interneurons, sensory afferents and/or descending bulbospinal axons)? A discussion of this would be strengthen the manuscript. The authors do present some interesting data on the “Peak-to-Peak Amplitude Across Time Bins”. This may suggest an effect on recruiting poly-synaptic connections, and you do provide some discussion of this question in the “Potential mechanisms of CLES-induced enhancement of evoked responses” paragraph of the Discussion section.

4.The authors should show representative traces from the other groups. Mainly since you present group data showing no change in amplitude over time.

5.Stimulus-response curves can be useful in detecting changes in cortical mapping - how does it relate to neural efficacy - about the respiratory network as presented in this study? Did the facilitation or increase in sMEP amplitude in CLES group translate to enhanced recovery as measured by EMG activity on the injured side? Or ventilation? The above information is important considerations in determining the value of this study to facilitate rehabilitative efforts using this technique. Other studies using stimulation or other modulatory efforts have shown enhancement of both the ipsilesional and contralesional side of the diaphragm; it is surprising that here the authors did not detect any changes in the contralesional diaphragm. Is the stimulation only targeting the ipsilesional motoneurons? What was the effect of this simulation at the cervical level on other muscles innervated by motoneurons within the cervical spinal cord?

6.Why did the authors perform the epidural stimulation at only the C4 level when the phrenic nucleus extends from C3 to C5? Do the authors have any data about the anatomical “spread” of the stimulation? Specifically, are PhMNs located rostral and/or caudal to the stimulation site being activated by the stimulation? Or do they have indirect evidence of this whereby subregions of the diaphragm innervated by C3 or C5 PhMNs are activated (as determined with their EMG recordings)? Given that the epidural stimulation is likely not inducing PhMNs (and/or other neurons part of the phrenic system) to fire action potentials - but is instead likely affecting the threshold for these neurons to fire. Thus, could the single stimulation site be sufficient to ultimately impact the firing of PhMNs across the entire phrenic nucleus on the side of stimulation (or even on the side contralateral t stimulation)?

7.The closed-loop stimulation was performed for 12-20 hours, without explaining why there was such a significant variation in simulation duration in this study. Did the injured rats that showed more substantial increases in sMEP get more prolonged stimulation, and how was the duration of the stimulation chosen?

8.What was the rationale for the three-day stimulation protocol, and did the authors examine for changes after the three days of stimulation? Why didn’t the authors continue to perform their analyses after the final day of stimulation? Did an increase in sMEP persist after the stimulation? This would be useful for assessing the potential persistence of an effect even after discontinuing stimulation. In addition, since they observe facilitation in the C2HS+CLES group over time from days 0 to 3, it would be interesting to know if this trend continues over the days after the stimulation is over. They perform their sMEP analysis each day after the epidural stimulation is over (i.e., on the same day as stimulation, but immediately after the stimulation paradigm is complete on that day), showing that the effect does persist as least for a relatively-brief amount of time; therefore, it wouldn’t be that surprising of an effect persisted even on subsequent days after the final day of epidural stimulation.

9.Finally, one reviewer noted that the authors need to provide a description of testing for software used here.

## Author Response

We sincerely thank the reviewers and the editor for their insightful and helpful reviews. We hope that we have adequately addressed the major concerns and believe this has strengthened the manuscript. See our responses below. We’ve also highlighted changes to the manuscript in yellow.

Major issues:

1.The study does not provide a comprehensive exploration of the approach and importantly does not demonstrate it is superior to the current open-loop stimulation approaches being explored by others. One reviewer expressed concern is that you have not shown that closed-loop stimulation is superior to open-looped stimulation as you predict. However in online discussion, we noted that you may be able to adequately address this in your discussion rather than necessarily insisting on new experiments. I suggest that you emphasize the limitations of the data presented and raise possibility for future studies to directly compare the two. Alternatively, if you have solid data comparing closed and open loop systems, you may want to consider including these.

a.We have expanded the limitations section in the discussion to reiterate why we think CLES could confer advantages vs open-loop stimulation paradigms but also clarified that we have not directly tested that hypothesis in this study. We state that further work is required to definitively draw conclusions regarding effectiveness of any particular stimulation strategy. We do have studies planned in this arena but this was outside the scope of the current paper.

2.Since the contralateral hemi-diaphragm is functionally intact after the C2 hemisection SCI, why did the authors not restrict the implantation of the epidural stimulating electrodes to only the phrenic nucleus ipsilateral to the injury while still using contralateral hemi-diaphragm activity as the trigger? They present differences in the SRS results between the ipsilesional and contralesional muscle sides, providing interesting data that suggest a potentially selective impact of the injured circuit on the effects of their closed-loop epidural stimulation paradigm. Therefore, there may be some experimental advantages to this bilateral stimulation design; this sided difference in SRS findings was just one example. What about translational considerations for the eventual implantation of such an approach? In general, a discussion of your rationale for the design of stimulation location(s) would be helpful.

a.We added another subheading in the discussion labeled, “Epidural Electrode Placement” whereby we have outlined our entire rationale for the C4 bilateral placement. In truth, these are the first longitudinal studies to explore epidural stimulation in freely-behaving rats for respiratory recovery and we relied upon published reports of anatomy (i.e. where was the highest proportion of phrenic motor neurons in the pool?) and phrenic motor plasticity (e.g. latent crossed pathways known for eliciting respiratory plasticity especially after upper cervical spinal cord injury as well as known mechanisms of phrenic motor neuron plasticity are located in this spinal region). We also added that further investigation is warranted to truly know the cellular mechanisms at play here as well as the potential for eventual translation (especially given our differential ipsi- vs. contralateral results).

3.At an anatomical/circuit level, do the authors believe the epidural stimulation is acting upon the PhMNs directly and/or indirectly on the PhMNs (for example, by activating pre-phrenic interneurons, sensory afferents and/or descending bulbospinal axons)? A discussion of this would be strengthen the manuscript. The authors do present some interesting data on the “Peak-to-Peak Amplitude Across Time Bins”. This may suggest an effect on recruiting poly-synaptic connections, and you do provide some discussion of this question in the “Potential mechanisms of CLES-induced enhancement of evoked responses” paragraph of the Discussion section.

a.We hope that the paragraph regarding rationale for the electrode placement mentioned above has adequately addressed this point as well. We included several possible substrates upon which CLES could be acting including: sensory afferents, pre-phrenic interneurons, cervical proprioceptive neurons, glia, or even ventral structures due to the low-resistivity of CSF.

4.The authors should show representative traces from the other groups. Mainly since you present group data showing no change in amplitude over time.

a.We have added representative traces from all four animal groups to Figure 2A and have updated the results section to reflect that.

5.Stimulus-response curves can be useful in detecting changes in cortical mapping - how does it relate to neural efficacy - about the respiratory network as presented in this study? Did the facilitation or increase in sMEP amplitude in CLES group translate to enhanced recovery as measured by EMG activity on the injured side? Or ventilation? The above information is important considerations in determining the value of this study to facilitate rehabilitative efforts using this technique. Other studies using stimulation or other modulatory efforts have shown enhancement of both the ipsilesional and contralesional side of the diaphragm; it is surprising that here the authors did not detect any changes in the contralesional diaphragm. Is the stimulation only targeting the ipsilesional motoneurons? What was the effect of this simulation at the cervical level on other muscles innervated by motoneurons within the cervical spinal cord?

a.The reviewers bring up extremely important points regarding the relationship between enhanced evoked potentials and behavior; in this case recovery of breathing function. We have added a paragraph outlining why we see our initial results of enhanced sMEPs as encouraging and how several other studies show that enhancement of evoked diaphragmatic potential amplitude is correlated with enhancement of functional outcomes (e.g. phrenic nerve output and plethysmographic ventilatory output). We do indeed have planned experiments to test these very questions in our model but it was outside the scope of this study.

6.Why did the authors perform the epidural stimulation at only the C4 level when the phrenic nucleus extends from C3 to C5? Do the authors have any data about the anatomical “spread” of the stimulation? Specifically, are PhMNs located rostral and/or caudal to the stimulation site being activated by the stimulation? Or do they have indirect evidence of this whereby subregions of the diaphragm innervated by C3 or C5 PhMNs are activated (as determined with their EMG recordings)? Given that the epidural stimulation is likely not inducing PhMNs (and/or other neurons part of the phrenic system) to fire action potentials - but is instead likely affecting the threshold for these neurons to fire. Thus, could the single stimulation site be sufficient to ultimately impact the firing of PhMNs across the entire phrenic nucleus on the side of stimulation (or even on the side contralateral t stimulation)?

a.We addressed these points in the “Epidural Electrode Placement” paragraph indicating that we do not expect a significant amount of “spread” of the current but that the majority of what we hypothesized as the interesting spinal substrates are located in the C4 region. As stated above, we also explain why we stimulated bilaterally for these initial experiments and postulated several ways we think this current could be acting to elicit plasticity.

7.The closed-loop stimulation was performed for 12-20 hours, without explaining why there was such a significant variation in simulation duration in this study. Did the injured rats that showed more substantial increases in sMEP get more prolonged stimulation, and how was the duration of the stimulation chosen?

a.We have added additional information to the “Chronic CLES and diaphragm EMG recording” subsection of the “Methods” section to quantify the distribution of stimulation duration. We added information explaining why this variation in stimulation duration occurred. We also added a line pointing readers to the “Collapsed Variables” subsection (which we moved from the “Results” section to the “Methods” section), which lists the variables that were not strictly controlled in this study, explains how we tested to determine if they affected our outcome measure of peak-to-peak amplitude, and states our ultimate decision to collapse these variables and pool the data because they did not have a significant impact.

8.What was the rationale for the three-day stimulation protocol, and did the authors examine for changes after the three days of stimulation? Why didn’t the authors continue to perform their analyses after the final day of stimulation? Did an increase in sMEP persist after the stimulation? This would be useful for assessing the potential persistence of an effect even after discontinuing stimulation. In addition, since they observe facilitation in the C2HS+CLES group over time from days 0 to 3, it would be interesting to know if this trend continues over the days after the stimulation is over. They perform their sMEP analysis each day after the epidural stimulation is over (i.e., on the same day as stimulation, but immediately after the stimulation paradigm is complete on that day), showing that the effect does persist as least for a relatively-brief amount of time; therefore, it wouldn’t be that surprising of an effect persisted even on subsequent days after the final day of epidural stimulation.

a.In truth, we used a 3-day stimulation protocol in order to test the initial hypothesis that CLES induces any sort of facilitation in the first place. Logistically, rather than extend the period of stimulation, we chose to end the study and publish our results. Our lab currently has several ongoing studies looking both at longer time periods of stimulation as well as how long the facilitation lasts after stimulation has stopped. Both of these parameters were outside the scope of the current study. We have also changed the title of the manuscript to remove the word “chronic” and to add “in freely behaving rats”.

9.Finally, one reviewer noted that the authors need to provide a description of testing for software used here.

a.We have added a subsection within the “Materials and Methods” section, which details all the software used in this project and the purpose that each software was used for. In that section, we also point readers to the “Experimental Design and Statistical Analysis” subsection, which expands on how statistical testing was performed.
